# Scientific Support by the BONUS+ Projects for the Sustainability of the Baltic Sea Region: The Case of the HELCOM Baltic Sea Action Plan

**DOI:** 10.1007/s13280-013-0472-9

**Published:** 2014-01-12

**Authors:** Kaisa Kononen, Andris Andrusaitis, Maija Sirola

**Affiliations:** BONUS Secretariat, Hakaniemenranta 6, 00530 Helsinki, Finland

**Keywords:** Baltic Sea, HELCOM, The joint Baltic Sea research and development programme, Linking science and policy, Sustainable ecosystem services

## Abstract

The synthesis of the BONUS+ research is introduced. The HELCOM Baltic Sea Action Plan is examined as a case to illustrate the potentials and challenges in building the science–policymaking interface on a macroregional level. The projects address environmental challenges in the Baltic Sea as defined by the Baltic Sea Action Plan, or consider the environmental governance and decision making within the Baltic Sea context in general. Eutrophication, biodiversity, hazardous substances, maritime activities, and the environment governance are addressed, as are crosscutting issues, such as the impact of climate change, maritime spatial planning and impacts of future development on ecosystem services. The projects contributed to relevant policy developments: 37 consultations carried out at EU level, 49 modifications to policy documents and action plans, 153 suggestions for the efficacy of pertinent public policies and governance, and in 570 occasions, scientists working in BONUS+ projects served as members or observers in scientific and stakeholder committees.

## Introduction

In 2006, the joint Baltic Sea research and development programme, BONUS, set its goal to create a cooperative, interdisciplinary, and transnational research program in support of the Baltic Sea region’s sustainable development. BONUS tasked itself to provide science support to policymaking, and in this way facilitated the implementation of ecosystem-based management of the environmental issues in the Baltic Sea region. The recent analysis (Andrusaitis et al. 2013) revealed around 80 various macroregional,^1^ EU and international policy initiatives to which the science output of BONUS could be potentially relevant. In this article, we introduce the synthesis of the BONUS+ projects that were implemented during 2009–2011 and that piloted the current research governance framework of BONUS. The HELCOM Baltic Sea Action Plan is also examined as a case to illustrate the potentials and challenges in building the science–policymaking interface on a macroregional level. The Baltic Marine Environment Protection Commission (HELCOM) is the central and the most mature transnational cooperation body in the field of environment in the Baltic Sea region and one of the pivotal stakeholders of BONUS. Through the activities of HELCOM, the scientific contribution of BONUS is mediated to a large number of other interlinked policy initiatives, such as the United Nations Convention on Biological Diversity, the EU Strategy for the Baltic Sea Region, the EU Marine Strategy Framework Directive, the EU Common Fisheries and Agriculture policies, and the VASAB Long-Term Perspective for the Territorial Development in the Baltic Sea Region, to name only a few.

### Ecosystem Approach to Management in the Core of BONUS and HELCOM Baltic Sea Action Plan

In September 2007, the key national research funding institutions under the respective ministries, governing research/education in nine states surrounding the Baltic Sea and the European Union, launched the first call for research proposals under the BONUS EEIG, referred to as the BONUS+ call (Anonymous 2007). The aim of the call was to fund research projects that focus “on supporting an ecosystem-based approach to management of human activities.” Two months later, in November 2007, the environmental ministers of the same nine countries adopted an agreement on implementation of the HELCOM Baltic Sea Action Plan, this also being fully based on the “ecosystem approach to management” (HELCOM 2007).

Although opening of the BONUS+ call and the adoption of the Baltic Sea Action Plan were important milestones of two independent processes that originated in different sectors, their parallelism is not a coincidence. On the one hand, the increasing societal impact of research funded at national and/or the European level has started to be recognized in various fora. Moreover, the need for a fundamental change in how the role of science is valued and what it can offer in the society is increasingly realized among the science policy authorities (e.g., European Commission 2009). On the other hand, the global policies dealing with ocean and coastal sustainability stress the importance of designing management actions based on the best scientific knowledge and identifying actions that support marine research, monitoring and evaluation, technology, and capacity transfer as one of their key objectives (IOC/UNESCO et al. 2011).

### Transnational and Interdisciplinary Calls to Solve Environmental Issues in the Region

The BONUS+ call, worth a total of EUR 22 million, was one of the many outcomes of the European Research Area process launched by the European Union in 2000. The European Research Area process was set out to increase transnational research cooperation by jointly defined research agendas and commonly funded research programs. Already during the 5 years of preparation under an earlier ERANET project, BONUS developed a joint Science Plan and Implementation Strategy (Hopkins et al. 2006). This plan identified seven broad research themes, which served as the basis of the BONUS+ call: (1) linking science and policy, (2) understanding climate change and geophysical forcing, (3) combating eutrophication, (4) achieving sustainable fisheries, (5) protecting biodiversity, (6) preventing pollution, and (7) integrating ecosystem and society. With the formulation of the call priorities, BONUS sent a clear message to the research community about focusing on interdisciplinary projects, and in particular, projects that integrate the societal research addressed in the themes (1) and (7) with natural research themes being featured predominantly in the other five themes (Anonymous 2007). In total, 148 letters of intent were received resulting in a very tight competition among the proposals. Importantly, the enormous interest that was generated by the call also provided a clear message of the willingness of the predominantly academic research community to contribute to solving the practical environmental issues of the Baltic Sea region. As an outcome of the BONUS+ call, 16 transnational proposals were selected for funding, and were subsequently implemented as research projects during 2009–2011. This special issue of *AMBIO* (Vol. 43, issue 1) provides an overview of the outcomes of 10 of these projects.

### HELCOM Baltic Sea Action Plan to Provide Comprehensive Environmental Roadmap Plan for the Region

The launch of the HELCOM Baltic Sea Action Plan was built on 30 years of experience with macroregional cooperation in protection of the Baltic Sea marine environment. When adopted in 2007 after an extensive preparatory work that spanned several years, the Baltic Sea region had for the first time a comprehensive environmental action plan that was explicitly based on the ecosystem approach. The Baltic Sea Action Plan set its goal to restore a good environmental status of the Baltic Sea by 2021 and complied to the following key elements: the recognition of the importance of integrated management of all human activities impacting on the marine environment, employment of the best available scientific knowledge about the ecosystem and its dynamics as well as identification and implementation of actions improving the health of the marine ecosystem, thus supporting sustainable use of ecosystem goods and services (HELCOM 2007).

The Baltic Sea Action Plan builds upon four mutually interlinked segments corresponding to four major environmental challenges in the Baltic Sea, namely (1) eutrophication, (2) hazardous substances, (3) biodiversity and nature conservation, and (4) maritime activities. In addition, it highlights two crosscutting methodological issues: developing assessment tools and methodologies and strengthening the governance and management through increased public awareness, credible cost–benefit analysis, improved cost–efficiency of measures and functioning funding mechanisms.

The novelty of the Baltic Sea Action Plan was and continues to be the translation of its qualitative goals into a set of clear and scientifically justified ecological objectives that are further operationalized through quantifiable indicators and targets (Backer and Leppänen 2008). Moreover, the plan envisions concrete measures based on the best available scientific knowledge about the relationships of the environmental pressures with dynamics and structure of the ecosystem, for example, a modeling-based nutrient load reduction targets allocated to the Baltic Sea countries (see, e.g., Wulff et al. 2007).

### BONUS+ to Contribute to Environmental and Sustainability Policy of Different Scales

In addition to providing scientific outputs supporting the implementation of the HELCOM Baltic Sea Action Plan, as summarized in this *AMBIO* issue, scientists of the BONUS+ projects participated actively in a number of national and international policy fora and contributed directly to various environmental and sustainability policy developments (Box 1).

**Box 1:** BONUS+ contribution to policy development. Source: the performance statistics of the BONUS+ projects

	Examples
37 contributions to consultations carried out at EU level	IBAM consortium was represented in Scientific, Technical and Economic committee for fisheries within the European Commission BALTICWAY contributed to the consultations on documents by the European Academies Scientific Advisory Council BALCOFISH advised to the EU Marine Strategy Framework Directive Task Group on quantitative descriptor 8: Contaminants and pollution effects ECOSUPPORT presented its model projections of the Baltic Sea to the European Parliament PREHAB took part in the European Commission consultation on maritime spatial planning and integrated coastal zone management
49 modifications to relevant policy documents and action plans on the Baltic Sea region and national level	BALCOFISH provided input to HELCOM Monitoring and Assessment Group on core indicators and indicator fact sheets concerning eelpout as an indicator species BEAST supported drafting of national reports for the EU Marine Strategy Framework Directive Descriptor 8 IBAM contributed to development of methodology used to calculate maximum sustainable yields for herring and sprat fisheries for International Council for the Exploration of the Sea (ICES) which was used as the basis of their recommendation to the European Commission RECOCA scientists contributed to the update of the HELCOM Baltic Sea Acton Plan nutrient reduction quotas ECOSUPPORT scientists contributed to the HELCOM 2013 thematic assessment of the climate change in the Baltic Sea area
153 suggestions for the design, implementation and evaluation of the efficacy of pertinent public policies and governance	PREHAB provided advice on mapping and protection of fish habitats in marine protected areas in 10 counties of Finland and Sweden ECOSUPPORT and RECOCA scientists presented a conceptual position article: “An outlook to the future Baltic Sea: how can we reach the targets of the Baltic Sea Action Plan?” at the joint final stakeholder conference of these projects BEAST presented recommendations for CORE and candidate bioeffect indicators for future HELCOM Monitoring and Assessment Group meetings on core indicators and revision of environmental targets BALCOFISH provided input to the revision of the Danish monitoring programme for nature and environment 2011–2015 concerning marine monitoring of contaminants and pollution effects AMBER results of the modeling efforts were taken into account in the plans of the Lithuanian Ministry of Environment on activities in the Nemunas river basin
In 570 occasions, scientists working in BONUS+ projects served as members or observers in scientific and stakeholder committees	ICES Working Group on Baltic Fisheries Assessment; Working Group on Biological Effects of Contaminants; Advisory Committee ICES/HELCOM Working Group on Integrated Assessments of the Baltic Sea HELCOM CORESET project committee for biodiversity HELCOM TARGREV group for reviews on ecological targets for eutrophication BACC II Science Steering group Curonian Lagoon Transboundary International Stakeholder Committee Steering Committee of the EU FP7 Deep Sea and Subseafloor Frontier Coordinated Action EU FP7 Environment and Climate Change Advisory Group

## Thematic Coverage of the BONUS+ Projects

**Fig. 1:**
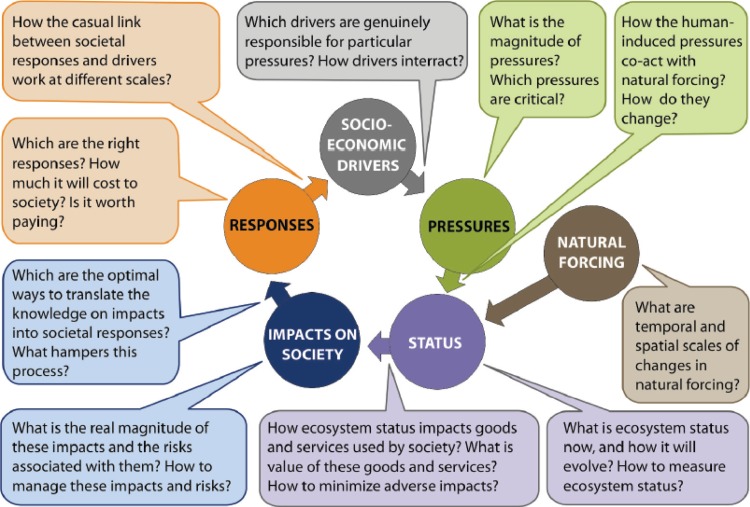
Some knowledge needs arising while implementing the framework of indicators distinguishing driving forces, pressures, status, impacts, and responses of the DPSIR framework of adaptive management (picture from Andrusaitis et al. 2013). Readers are invited to compare this cycle with visualizations of other related cyclic processes presented in Figs. 1 and 5 of Meier et al. 2014 (this volume)

The thematic coverage of the BONUS+ call was very broad, and the actual selection of the projects to be funded was based on the scientific excellence without predefined earmarking for any of the themes. In such a situation, there is always a risk that the selection of projects becomes thematically biased: the priority in selection may be given to well-developed areas that were viewed more capable to produce excellent research proposals, while much needed knowledge from less-developed areas is overlooked, this despite both being equally needed for well-informed policy and management actions. Interdisciplinary research proposals in areas like environmental socioeconomy and environmental policy are often awarded lower evaluation scores in comparison with more traditional natural science proposals. In contrast, when the well-known “drivers–pressures–status–impact–response” (DPSIR) adaptive management circle (e.g., Atkins et al. 2011) is used as a vehicle generating the critical research questions (Fig. 1), it becomes apparent that the most complicated questions originate from the sector representing the complex societal realm (e.g., Andrusaitis et al. 2013).

In spite of this risk, the funded BONUS+ projects eventually addressed all the four major environmental challenges in the Baltic Sea as defined by the Baltic Sea Action Plan, or considered the environmental governance and decision making within the Baltic Sea context (Table 1).

**Table 1:** Synergies among the BONUS+ research themes and the segments and crosscutting issues of the Baltic Sea Action Plan. Names in the cells are the acronyms of 16 BONUS+ projects. Results of the projects noted in brackets are not presented in this *AMBIO* issue

BONUS+ research themes	HELCOM Baltic Sea Action Plan segments and crosscutting issues					
	Eutrophication	Hazardous substances	Biodiversity and nature conservation	Maritime activities (incl. maritime spatial planning)	Monitoring and assessment	Governance and decision making
1. Linking science and policy 7. Integrating ecosystem and society	**RECOCA AMBER IBAM PROBALT**	**(BALCOFISH) BEAST IBAM**	**IBAM**			**(AMBER) PROBALT (RISKGOV)**
2. Understanding climate change and geophysical forcing	**AMBER (B-GAS) BALTIC-C ECOSUPPORT INFLOW**			**BALTICWAY**	**BALTIC-C**	
3. Combating eutrophication	**(AMBER) (B-GAS) BALTIC-C ECOSUPPORT INFLOW RECOCA**				**(AMBER) BALTIC-C**	**RECOCA IBAM**
4. Achieving sustainable fisheries						**IBAM**
5. Protecting biodiversity			**(BAZOOCA) PREHAB (BALTGENE)**	**PREHAB**	**PREHAB**	**PREHAB**
6. Preventing pollution		**(BALCOFISH) BEAST**			**(BALCOFISH) BEAST**	

Eutrophication was addressed by the majority of the projects; in total eight projects, biodiversity by four, hazardous substances by three, and maritime activities (including maritime spatial planning) by two BONUS+ projects. The Baltic Sea environment governance and the decision making were addressed by six projects. The crosscutting issues, such as the impact of climate change, were studied by three projects, maritime spatial planning by two and impacts of future development on Baltic Sea ecosystem services by three projects.

### Combatting Eutrophication in the Changing Climate

Eutrophication is commonly considered as the most severe environmental problem in the Baltic Sea, and therefore, the most important component of the Baltic Sea Action Plan. The project RECOCA was the successor of a research effort commenced already in 1999 by the Baltic Nest Institute and its predecessors aiming at developing a decision support tool for nutrient abatement measures. The project has through its different phases progressively developed a modeling tool for estimating various options in nutrient loading and abatement measures, and their economic implications. In BONUS+, RECOCA focused on processes in the catchment and used a multimodel approach to characterize the nutrient loads, the retentions that occur between these sources and the sea, and the effects of various management strategies to reduce loads (Wulff et al. 2014). The project produced estimates of the needed reductions in nutrient loading from different catchments of the drainage basin to reach the reduction goals as formulated by the Baltic Sea Action Plan. Furthermore, the project estimated the impacts of various abatement measures on nutrient loads into the sea, as well as analyzed the capacity of the Baltic Sea countries for implementing the different measures in different parts of the catchments. As a summary outcome, the project concluded that the Baltic Sea Action Plan load reduction targets can be reached in all subregions of the Baltic Sea except for nitrogen reductions in the Danish Straits and phosphorus reductions in the Baltic Proper. Noteworthy is, however, that due to high retention of nutrients in the drainage basin, more than eight times more nutrients would need to be reduced within the catchments compared with the set reduction target of nutrient loading into the sea.

Among the disputed options requiring efficient transnational policy are, for example, sharing the costs of nutrient abatement across national borders or introducing nutrient emission trading. Findings of this international team of scientists mark the future pivotal directions for successful implementation of the Baltic Sea Action Plan.

It is obvious that implementation of the current environmental and sustainability policies, such as the Baltic Sea Action Plan, will occur under the impact of climate change. Five different BONUS+ projects (HYPER, ECOSUPPORT, AMBER,^2^ BALTIC-C, and INFLOW) applying different approaches attempted to project the impact of climate change on the Baltic Sea environment (Carstensen et al. 2014; Meier et al. 2014; Omstedt et al. 2014; Kotilainen et al. 2014). The common concept of “understanding the past to model the present and predict the future” unites all these projects.

The crucial impact of oxic/hypoxic/anoxic interplay in the Baltic Sea bottom layers was demonstrated by the project HYPER (Carstensen et al. 2014). The project enriched the scientific basis for further implementation of Baltic Sea Action Plan with quantitative knowledge of nutrient release and recycling processes under various environmental conditions. Thus, it is now possible to assume with high level of confidence that due to transformation of buried organic phosphorus into reduced Fe-phosphate minerals, rapid oxygenation of the anoxic bottom (as proposed by the proponents of geoengineering) might lead to even more massive internal loading of phosphorus if the sediment becomes anoxic again. Biologists involved in the HYPER project were able to demonstrate a relationship between the oxygen conditions and the benthic community’s capacity in providing important ecosystem services that might be impaired even by recurrent brief episodes of oxygen deficiency.

By testing future scenarios for the first time with an ensemble of three different biogeochemical models, project ECOSUPPORT (Meier et al. 2014) was able to demonstrate the increasing importance of nutrient load reduction and sustainable fisheries management in conditions of future climate. The targets currently set within the Baltic Sea Action Plan will most possibly have to be tightened. Moreover, because of the regional differences of climate change impact, current proportion of the nutrient load reduction among the Baltic Sea countries will most probably have to be revisited in future. Noteworthy, one of the deficiencies in our predicting capacity pinpointed by the ECOSUPPORT multimodel approach—understanding of the nutrient filtering properties of the coastal zone—was effectively addressed by another BONUS+ project: AMBER. The AMBER scientists were able to show the crucial importance of the nitrogen removal taking place in shallow (down to 20–25 m) coastal waters. On the other hand, climate change can increase loadings of terrestrial organic nitrogen to some parts of the Baltic Sea: a significant nutrient source for phytoplankton and bacteria (Voss et al. 2011; Korth et al. 2012). Loading of organic substances that have hardly been monitored in the past deserve close attention while designing the future monitoring programs.

This statement is indirectly supported also by the BONUS+ project BALTIC-C. By building a carbon budget based on exceptionally detailed observations of dissolved and atmospheric CO_2_, the scientists of this project discovered that during the later phase of the phytoplankton spring bloom in the central Baltic, the community sustains positive net biological productivity a certain time after the nitrate reserve is depleted. One of the hypothetic nitrogen sources sustaining net productivity during this period could be dissolved organic nitrogen. While analyzing past and present variations and projecting possible future changes in the Baltic Sea acid–base (pH) and oxygen balances, the BALTIC-C scientists used numerical experiments and a catchment–sea-coupled model in three scenarios: business as usual, medium scenario and the Baltic Sea Action Plan (Omstedt et al. 2014). These were combined with the scenarios A2 and B1 of the Intergovernmental Panel on Climate Change (IPCC 2007). Although the implementation of the Baltic Sea Action Plan will likely lessen the hypoxia and anoxia, the climate change and land-derived changes are projected to affect the Baltic Sea by amplifying the seasonal cycles in acid–base balance of water and alternating the deep-water conditions. A conclusion of utmost importance for policymaking is that if the Baltic Sea Action Plan’s nutrient reduction targets are not achieved, the impact of acidification and deep-water oxygen depletion in the future Baltic are likely to be much more severe.

Scrupulous reconstruction of the past hypoxia and anoxia episodes that have taken place during the whole span of geological history of the Baltic Sea and establishing their relationships to the past regimes of temperature and inflows of saline water allowed the scientists of INFLOW project (Kotilainen et al. 2014) to come to a very similar conclusion. They argue that the nutrient loads need to be further reduced in the future to minimize the effect of sea surface-temperature changes.

Co-acting with eutrophication, the projected alternation of climatic conditions might stimulate anaerobic degradation of organic matter buried in the sediment. As a consequence, more free methane gas will accumulate in sediment; this might eventually lead to enhanced escape of methane into the water column and consequently into atmosphere where it acts as a very potent greenhouse gas. Detailed investigation of the methane “hot spots” in the Baltic allowed the BALTIC GAS^3^ project scientists to conclude that the robust barrier to methane emission in the present climatic conditions, mainly in a form of sulfate-saturated sediment layer, may weaken with continued eutrophication and, warming and anoxia thereby enhancing the upward migration of gas.

### Understanding the Effects Hazardous Substances in the Baltic Sea Ecosystem

One of the goals of the Baltic Sea Action Plan is to render the Baltic Sea life undisturbed by hazardous substances. There is a general agreement that most often marine life is disturbed by a combined impact of many hazardous substances and other stressors. However, due to the gap of knowledge about relationships between measured concentrations of chemicals and their biological effects at different biological levels of organization, the traditional single-substance and concentration-based approach dominates in the assessment methodology.

Compared with many other seas and oceans, however, the amount of data and understanding of the biological effects of hazardous substances in the Baltic Sea have been scarce. Therefore, also the confidence to apply such information for monitoring and assessment of the status of ecosystem health has been lacking.

As a result of analyzing a wide range of biological effects of a number of hazardous substances by novel and already established methods, the project BEAST (Lehtonen et al. 2014) created scientific foundations for including four biological effects of contaminants: lysosomal membrane stability, fish diseases, micronuclei test and malformed eelpout and amphipod embryos, into the set of “pre-core” indicators for hazardous substances and their effects in the Baltic Sea (HELCOM 2013). Jointly with another BONUS+ project, BALCOFISH,^4^ an integrated biomarker assessment tool (IBAT), was created allowing for comparison of input data for measured biological effect parameters for which assessment criteria have been developed. The BonusHAZ database, also created through the collaboration of BEAST and BALCOFISH, is now the most comprehensive data collection on biological effects of hazardous substances in the Baltic Sea region. Among other important outcomes of the BEAST work is the significant advancement toward the harmonized environmental assessments of different sea areas in Europe, which is one of the objectives of the EU Marine Strategy Framework Directive.

### Scientific Support for Maritime Spatial Planning

As a response to intensifying and multifaceted uses of the Baltic Sea space, and concurrently with the start of the Baltic Sea Action Plan in 2007, HELCOM adopted Recommendation 28E/9 on development of broad-scale maritime spatial planning principles in the Baltic Sea area (HELCOM 2007). Moreover, following the request of the ministerial declarations of VASAB in October 2009 (VASAB 2009) and HELCOM in May 2010 (HELCOM 2010a), the joint HELCOM-VASAB working group was set up to ensure cooperation and coherent regional maritime spatial planning among the countries in the region and tackle the acute need for both general methodological research as well as more bespoke knowledge to fill in gaps in spatial information (HELCOM 2010b).

The theme “Linking science and policy” of the BONUS science plan and implementation strategy (Hopkins et al. 2006) listed both advancing integrated coastal zone management and maritime spatial planning among the target areas for development and application of the ecosystem approach to management. Two BONUS+ projects, PREHAB, and BALTICWAY, contributed to the strengthening the scientific basis for the maritime spatial planning.

One of the principal challenges for maritime spatial planning is scarcity of information on the spatial distributions (of necessary breadth of coverage and degree of resolution) of different species and habitats. To fill in the data gap, predictive methods that are based on statistical species–environment relationships, also called habitat- or species-distribution models, are often used. PREHAB evaluated applicability of habitat- or species-distribution models for predictive mapping across the Baltic Sea (Lindegarth et al. 2014). In order to achieve the highest level of predictability, PREHAB recommended to apply an ensemble approach, integrating the results of several specified methods for predictive mapping and assessing uncertainties of spatial patterns. Moreover, the PREHAB scientists assessed the effect of successful implementation of the Baltic Sea Action Plan on bottom vegetation habitat distribution and the reproduction areas of two commercially important fish species, and finally estimated the monetary benefits associated with the implementation of the Baltic Sea Action Plan in two Baltic coastal habitats.

Applying a very different approach, the BALTICWAY project examined how to make shipping as well as offshore and coastal engineering infrastructures environmentally safer by utilizing the new knowledge on semipersistent surface currents and the cutting-edge capacity to model these currents (Soomere et al. 2014). The BALTICWAY concept presumes that the risk of transport of probable environmentally harmful spills to vulnerable coastal areas could be minimized or at least the time for the response operations could be stretched longer. This could be achieved by considering the statistical distribution of the surface currents while routing maritime transportation and selecting location of engineering infrastructures. When developed further, this approach has the potential to produce science-based advice to practical decision-making regarding safer location of maritime activities. This in turn works toward reducing the environmental risks, thus contributing to maritime spatial planning and the maritime activities segment of the Baltic Sea Action Plan.

### Less-Known Issues in Protection of Biological Diversity

There are several areas within the otherwise “information-saturated” Baltic Sea Action Plan segment of biological diversity that are much less investigated, although their potential importance might be enormous. One of these areas is the genetic diversity within the populations of biota of the Baltic Sea. A significant contribution to the knowledge in this area was made by the project BALTGENE^4^ that simultaneously with some important methodological development was able to demonstrate a relationship between the genetic diversity within populations and healthy functioning of an ecosystem and its resilience. BALTGENE also collected new evidence on the real connectivity of subpopulations and the role of connectivity in securing sustainability of populations. This is supported also by the finding of the earlier mentioned BALCOFISH project that found the real genetic connectivity within the eelpout (*Zoarces viviparous* (L.)) population to be higher than what was commonly believed. These findings are crucial in, for example, establishing a coherent network of marine protected areas in the Baltic Sea as envisioned by the Baltic Sea Action Plan.

Another rapidly emerging study area relevant for the biological diversity segment of the Baltic Sea Action Plan applies to the food-web effects of biological invasions (see e.g., Kotta et al. 2006). The project BAZOOCA^5^ addressed one of the most challenging questions—potentially cascading effects of an invader comb jelly *Mnemiopsis leidyi*. Much concern about the effect of *Mnemiopsis* invasion was created due to its dramatic impact on the Black Sea ecosystem, difficulty it poses for traditional zooplankton monitoring as well as insufficient knowledge on its biology and ecology in the Baltic. The evidence collected by BAZOOCA brings in mostly good news: while the reproduction capacity of *Mnemiopsis leidyi* is insufficient under the low salinities that are characteristic of the Bornholm Basin, no self-sustaining population of *Mnemiopsis* was found in the Baltic proper—they are dependent on drift recruitment from more saline reproduction areas. Also, their abundance and predation was found too low to affect the zooplankton community. Although it may compete with larval cod for zooplankton, *Mnemiopsis* constitutes no direct threat to the Baltic cod as predator of eggs and larvae (Jaspers et al. 2011).

### Governance and Decision-Making in the Multinational Context

Using the best available scientific knowledge is in the core of the ecosystem approach to management, and therefore communication between the scientific community and decision makers is essential in bridging the scientific information and management actions. The project PROBALT considered the ensemble of various, both public and private, cooperative arrangements on national, macroregional and European levels of administrative scales that aim at managing the Baltic Sea environment, with a particular focus on mitigation of eutrophication (Tynkkynen et al. 2014). According to the results of the project, the main challenges are (i) the differences between coastal countries in terms of environmental conditions, including environmental awareness, (ii) overlaps of policies at different geopolitical levels (EU, macroregion, national, and subnational), (iii) the lack of adequate spatial and temporal specification of policies, and (iv) insufficient policy integration across sectors. Although the role of nongovernmental actors was not in the core of this study, PROBALT was able to pinpoint to the crucial importance of communication with and engagement of the variety of stakeholders to not only legitimize the governance actions but also to better specify the policies, both temporally and spatially. In this context the assets of knowledge communication are emphasized: without adequate knowledge about the problem, putting pressure and communicating various perceptions of the problem and possible solutions become impossible for the wider public.

The importance of the issue of stakeholder engagement is emphasized also by the RISKGOV^6^ project focusing on the risk management in five different thematic areas relevant to the Baltic Sea Action Plan: eutrophication, overfishing, invasive alien species, chemical pollution, and oil spills. These areas differ substantially in terms of complexity of sources of pressure, potential impact, the state of scientific knowledge, and uncertainty as well as the type and extent of sociopolitical ambiguity (Linke et al. 2013; Udovyk and Gilek 2013). As one of the key challenges, RISKGOV identified the necessity to make decisions in conditions of uncertainty. This implies to the importance of embracing the probabilistic character of knowledge, assessing the uncertainty and communicating it.

The topic on uncertainty of knowledge was also addressed in the IBAM project. IBAM developed a decision model based on the Bayesian probabilistic approach that considers both the existing scientific information and the experts’ beliefs. The model can be continuously updated with new information, thus narrowing uncertainty of its outputs. The examples studied concerned eutrophication as well as herring stock dynamics and fishery in the Gulf of Finland. Although relatively theoretic at its current level of development, this approach to management of the sustainability issues of the Baltic Sea ecosystem services has the capacity to provide decision makers with estimates of the potential effectiveness of management actions including the probability of success. Therefore, decisions can be made while understanding the level of risk associated with it (Rahikainen et al. 2014).

## Conclusions and Future Outlook

All BONUS+ projects have now finished their research, analyzed their results and published them in this *AMBIO* Special Issue and in many other fora. Today, six years after the launch of the BONUS+ call and adoption of the Baltic Sea Action Plan, it has become self-evident that these two independent events launched a unique process of linking science to policy, which has no analogy worldwide.

In terms of the future development of the Baltic Sea Action Plan, an emerging conclusion from the BONUS+ projects is the need for adaptive management. In such a management approach, the decision-making is based on progressively increasing scientific knowledge and the related action plans on iterative adjustments, taking into account spatial and temporal scales and differences, the level of uncertainty as well as socioeconomic developments.

Since finishing the BONUS+ programme, BONUS has launched the next Baltic Sea research and innovation call in late 2012 to fund further transnational and interdisciplinary projects. Acting as backbone for that call is the new strategic research agenda that has been created in close consultation with HELCOM and builds on the experience of the BONUS+ call. With it, the successive BONUS calls are becoming more targeted on the key sustainability issues of the Baltic Sea ecosystem services. In this way BONUS scientific support to ensure the implementation of ecosystem approach to management through the HELCOM Baltic Sea Action Plan will grow even stronger.

## Footnotes

1 Addresses common challenges faced by a defined geographic area relating to countries located in the same geographic areas, for example, the Baltic Sea region, which thereby benefit from strengthened cooperation contributing to achievement of economic, social, and territorial cohesion.

2 AMBER project is not presented in this volume.

3 BALTIC GAS project is not presented in this volume.

4 BALCOFISH project is not presented in this volume.

5 BALTGENE and BAZOOCA projects are not presented in this volume.

6 RISKGOV project is not presented in this issue.
